# Reconstruction of lateral coherence and 2D emittance in plasma betatron X-ray sources

**DOI:** 10.1038/s41598-024-52231-z

**Published:** 2024-01-19

**Authors:** Alessandro Curcio, Alessandro Cianchi, Gemma Costa, Alessio Del Dotto, Francesco Demurtas, Massimo Ferrario, Maria Dolores Rodríguez Frías, Mario Galletti, José Antonio Pérez-Hernández, Giancarlo Gatti

**Affiliations:** 1 INFN-LNF, via Enrico Fermi 40, 00044 Frascati, Rome Italy; 2 Department of Physics, Università di Roma Tor Vergata, Via Ricerca Scientifica 1, 00133 Rome, Italy; 3 INFN-Tor Vergata, Via Ricerca Scientifica 1, 00133 Rome, Italy; 4 NAST Centre, Via Ricerca Scientifica 1, 00133 Rome, Italy; 5 Centro de Laseres Pulsados (CLPU), Edificio M5, Parque Científico, C/ Adaja 8, 37185 Villamayor, Salamanca Spain; 6 Dpto. Física y Matemáticas, Universidad de Alcalá, Plaza de San Diego, s/n Alcalá de Henares, Madrid, Spain

**Keywords:** Applied physics, X-rays

## Abstract

X-ray sources have a strong social impact, being implemented for biomedical research, material and environmental sciences. Nowadays, compact and accessible sources are made using lasers. We report evidence of nontrivial spectral-angular correlations in a laser-driven betatron X-ray source. Furthermore, by angularly-resolved spectral measurements, we detect the signature of spatial phase modulations by the electron trajectories. This allows the lateral coherence function to be retrieved, leading to the evaluation of the coherence area of the source, determining its brightness. Finally, the proposed methodology allows the unprecedented reconstruction of the size of the X-ray source and the electron beam emittance in the two main emission planes in a single shot. This information will be of fundamental interest for user applications of new radiation sources.

## Introduction

Betatron X-ray sources based on plasma accelerators offer compact solutions for secondary sources of high-photon energies^1–8^. The peak brightness of these sources is comparable to the average brightness of existing synchrotron machines. Synchrotrons and plasma-driven betatron sources share the fact of being temporally incoherent, unlike the X-ray Free Electron Lasers^9^. The real uniqueness of plasma-based X-ray betatron sources lies in the shortness of the X-ray pulses, which reach the femtosecond scale^10–12^. At such extreme time scales, the ultrafast dynamics of atoms and molecules can be studied by time-resolved imaging^13–15^ and/or spectroscopy^16,17^. Concerning the first diagnostic technique, it has been generally accepted and demonstrated that betatron sources are laterally coherent, thus providing an efficient phase contrast^13,14,18–20^. A rough estimation of the lateral coherence has been performed in the past, exploiting the visibility of sharp edges, but this provided information on resolution for phase contrast imaging applications or source size rather than the lateral coherence function. Those results were in agreement with 1D synchrotron-like models of betatron radiation^21^. However, for a precise understanding of the degree of lateral coherence of betatron X-ray sources, a 3D approach to the electron dynamics and radiation seems essential. In this work, we observe that the lateral coherence of the betatron X-ray sources is determined by the breaking of the azimuthal symmetry imposed by the electron trajectories in the plasma focusing channels^22–24^. The spatial phase of the radiation field is modulated by the betatron dynamics and by the acceleration, imprinting nontrivial spectral-angular correlations to the photon beams, that we detected. Such an observation is based on a highly-resolved experimental measurement of the spectral-angular distribution of the betatron X-ray source. Indeed, the spectral-angular distribution of the source is strictly related to its lateral coherence. This can be understood by studying the so-called mutual coherence function $\Gamma (\Omega _1,\Omega _2,\tau )$, which is a measurement of the degree of local autocorrelation of a radiation field $\vec {H}(\Omega , t)$, defined as:

1 $$\begin{aligned} \Gamma (\Omega _1,\Omega _2,\tau )=\frac{\int dt \vec {H}^{*}(\Omega _1,t) \cdot \vec {H}(\Omega _2,t+\tau )}{\sqrt{\int dt |H (\Omega _1,t)|^2}\sqrt{\int dt|H (\Omega _2,t)|^2}} \end{aligned}$$

In Eq. (1), $\Omega _1$ and $\Omega _2$ are two different solid angles of observation, *t* and $\tau$ are observation times. In the following we’ll individuate a solid angle $\Omega$ via a polar angle $\theta$ and an azimuthal angle $\phi$, recalling the definition of unitary solid angle $d\Omega =d\phi d\theta \sin {\theta }$. The choice of adopting angles (with respect to the source point) instead of positions (at the detector plane) for the evaluation of the field across its transverse profile, orthogonal to the propagation axis, is motivated by the assumption of far-field. In this paper, the betatron radiation is measured in far-field conditions. Therefore, all the considerations are made in the same working frame. Specifically, the radiation field $\vec {H}$ denotes the magnetic component. In far-field conditions, the electric and magnetic field components of the radiation wave are related only by constants. Therefore, defining the mutual coherence function (Eq. 1) by means of one or the other component is fully equivalent. Furthermore, Eq. (1) can be rewritten in terms of the Fourier transform of the radiation field, using the convolution and the Parseval’s theorems:

2 $$\begin{aligned} \Gamma (\Omega _1,\Omega _2,\omega )=\frac{ \vec {H}^{*}(\Omega _1,\omega ) \cdot \vec {H}(\Omega _2,\omega )}{\sqrt{\int d\omega |H (\Omega _1,\omega )|^2}\sqrt{\int d\omega |H (\Omega _2,\omega )|^2}} \end{aligned}$$

Equation (2) is also known as lateral coherence function. It states that the lateral coherence is related to the spectral-angular distribution of the radiation source, expressed by $\vec {H}(\Omega ,\omega )$, where $\omega$ is the spectral frequency of the radiated field. Eventually, in this work we adopted a generalized definition of the lateral coherence function suitable for broad radiation spectra, such as the betatron radiation spectra from plasma accelerators, which is an average of Eq. (2) over all the emitted photons:

3 $$\begin{aligned} \Gamma (\Omega _1,\Omega _2)=\frac{\int d\omega \vec {H}^{*}(\Omega _1,\omega ) \cdot \vec {H}(\Omega _2,\omega )}{\sqrt{\int d\omega |H (\Omega _1,\omega )|^2}\sqrt{\int d\omega |H (\Omega _2,\omega )|^2}} \end{aligned}$$

Thus, starting from the spectral-angular distribution of the broadband source, it is possible to retrieve information on the lateral coherence, via Eq. (3).

**Figure 1:**
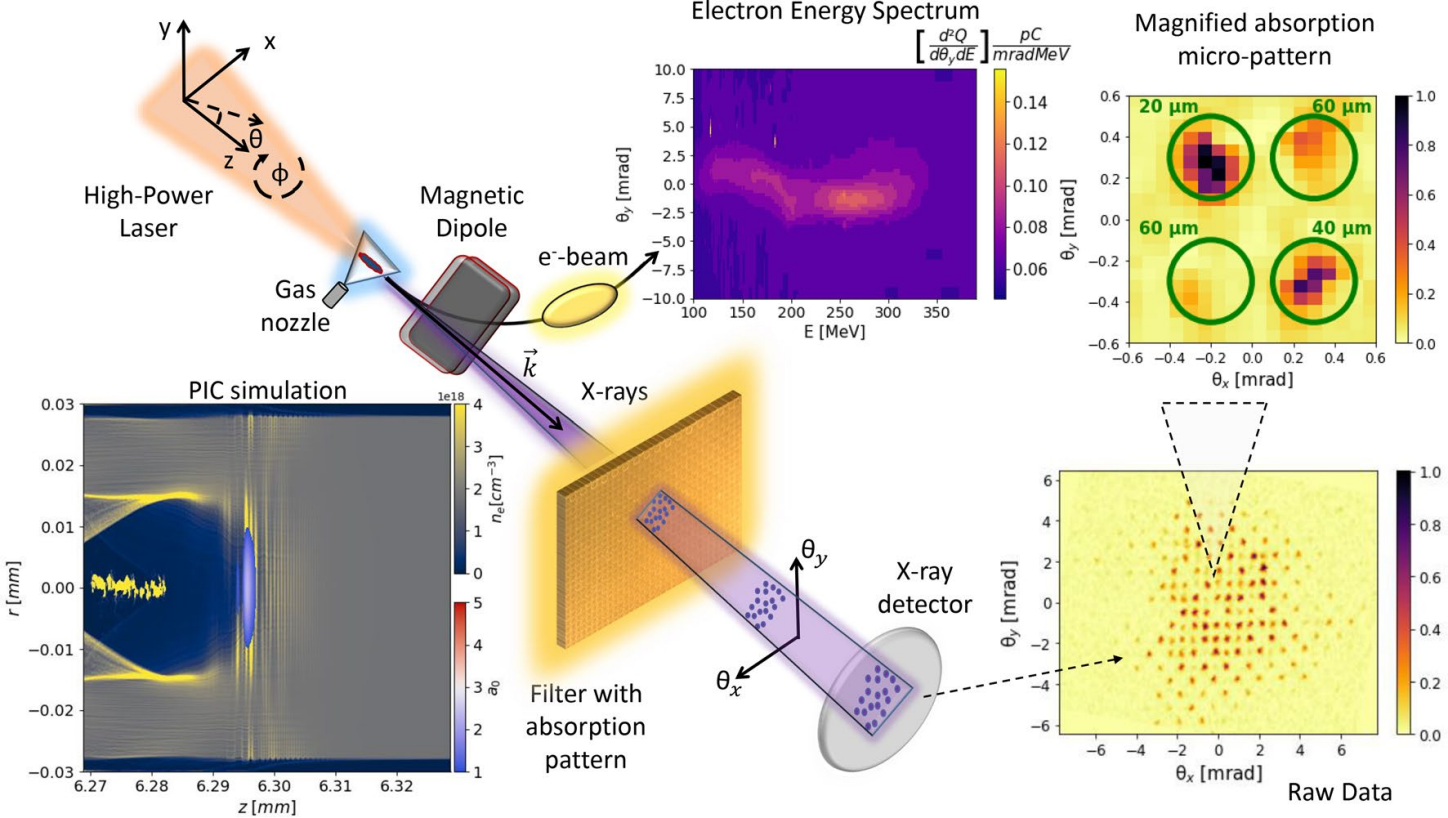
Experimental setup and geometry for characterization of the lateral coherence of the plasma-based betatron X-ray source. The observation angles are related with the polar coordinates via $\theta _{x}=\theta \cos {\phi }$ and $\theta _{y}=\theta \sin {\phi }$.

In this paper, we show how to perform a reconstruction of the lateral coherence function, based on the measurement of the spectral-angular distribution of the radiated energy. The method is not based on interferometric techniques, but on an optimized model of the phase and amplitude of the radiation field. The spectral-angular distribution is measured via a fit, using a standard technique in X-ray spectroscopy, where, from the pattern provided by a bunch of absorbers, the incident radiation spectrum is measured through a fit, yielding the spectral amplitude. In this work, the aim was to measure the spectral-angular distribution of radiation, which, for plasma betatron sources, is determined both by the field amplitude and phase. The best fitting radiation field was calculated for each observation angle via a model of betatron radiation in the wiggler regime including both 3D particle motion and acceleration.

**Figure 2:**
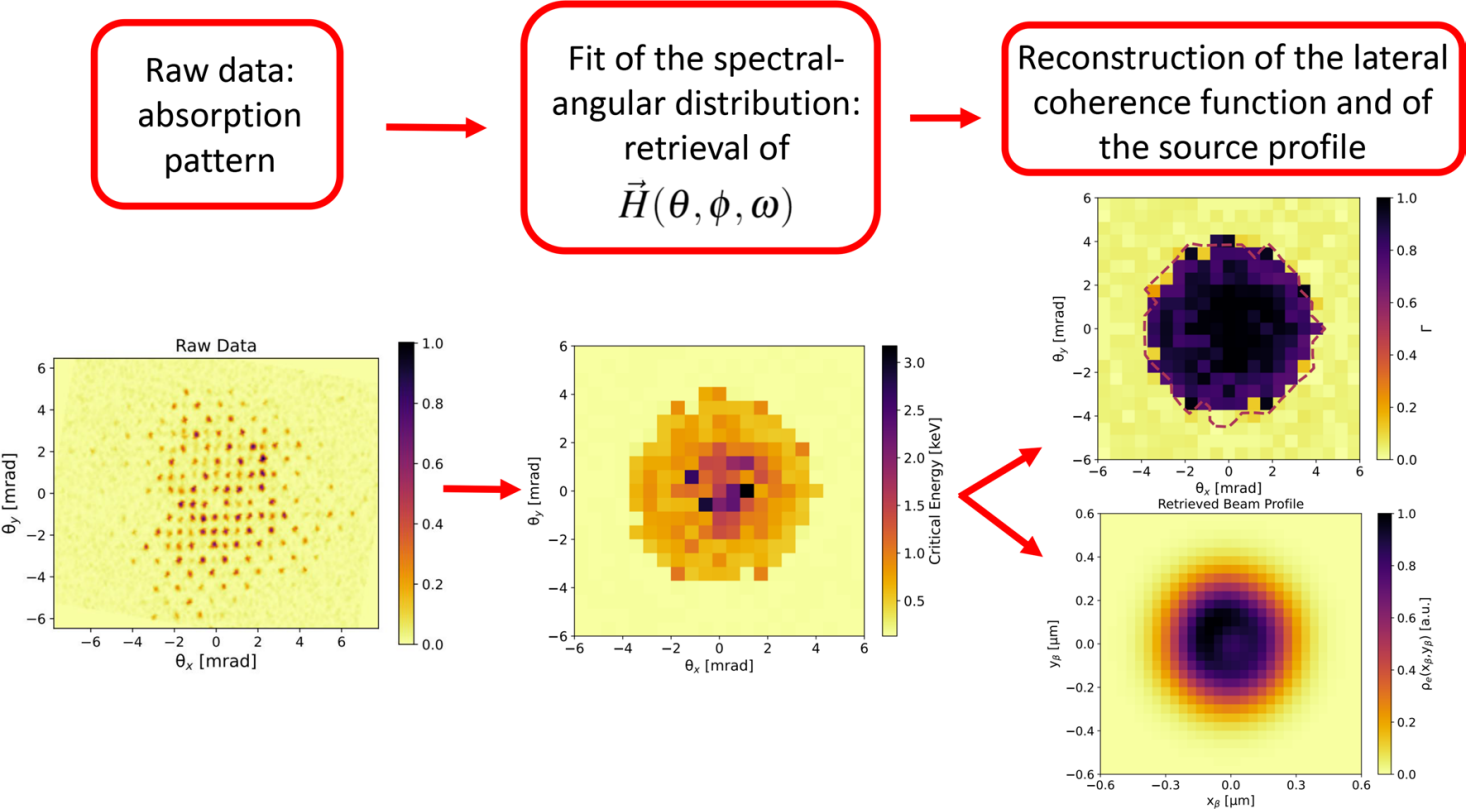
Flow chart of the procedure adopted in this paper, for the reconstruction of the lateral coherence function and of the X-ray source profile. From left to right: the raw data (left figure) shows an absorption pattern. For each observation angle (each identified by a micropattern that repeats across the absorption mask), a radiation field is calculated having the spectral properties to locally fit the measured absorption pattern. The best fitting radiation field enables the measurement of the spectral-angular distribution of the X-ray source (central figure). From the spectral-angular distribution of the X-ray source, the lateral coherence function is retrieved (top-right figure), by means of its definition in terms of the radiation field (Eq. (3)). Moreover, being the spectral-angular distribution of the radiation field related to the transverse profile of the X-ray source, the latter can also be reconstructed (bottom-right figure).

The purpose of our work has been to reveal the subtle details that characterize next-generation photon sources for users. One of them will be realized as a result of the EuPRAXIA Advanced Photon Sources (EuAPS) project^25^. To perform the experiment, we exploited the betatron X-ray source based on the compact laser-plasma accelerator at VEGA 2 (CLPU, Spain)^26^, in the setup depicted in Fig. 1. For the X-ray diagnostics, we used an absorption pattern between the source and the detector which allowed for spectral-angular resolution. In fact, the betatron radiation was transmitted through a micropatterned filter placed at 0.6 m from the source, which was an array where each element corresponded to a set of different thicknesses of Mylar, ranging from 20 $\upmu$m to 60 $\upmu$m (see top-right inset of Fig. 1). Each of the matrix element was placed at a specific observation angle from the source. In this way, the solid angle was sampled with a resolution of 1 $\upmu$sr. An Imaging Plate (IP) BAS-MS was used as a detector, wrapped in a 10 $\upmu$m thick Al foil and placed at 0.8 m from the source. For each observation angle, we fitted the measured transmitted pattern with the one expected from a betatron radiation source characterized by the plasma, electron and laser parameters of our experiment. The expected transmission pattern was obtained from a best fitting radiation field, characterized by both amplitude and phase. Minimizing the difference between the measured and expected patterns, allowed us to obtain the spectral-angular distribution of the betatron X-ray source in a single shot. Afterwards, we reconstructed the lateral coherence function, based on the spectral-angular distribution of the source, using Eq. (3). Finally, through the same minimization algorithm used for the fit, we also reconstructed the transverse profile of the X-ray source in the two main emission planes simultaneously, something otherwise impossible with a 1D approach^27–30^. Indeed, as discussed in this paper, the spectral-angular distribution of radiation from a plasma betatron source is related to the transverse profile of the latter via the spatial phase of the radiation field. Figure 2 shows a flow chart of the procedure followed in this paper, a process leading from raw data to radiation and electron beam parameters.

Fundamental aspects for the implementation of the proposed methodology were the use of a 3D model of betatron radiation, shown in the “Methods” section (“Theoretical background” subsection), which was more suitable for the case under study than a 1D synchrotron-type model, and the development of a numerical code to extract information from the transmission map provided by the raw data of the type in Fig. 2. A validation of the code used for the data analysis, shown in the “Methods” section (“Validation with synthetic data” subsection), was performed prior to the experiment, demonstrating the possibility to retrieve precise information on the spectral-angular distribution of the radiation field and also on the lateral profile of the X-ray source, equivalent to the electron beam profile. This eventually enabled the unprecedented characterization of transverse electron beam emittance in two planes simultaneously, an achievement that may have a strong impact for the electron beam diagnostics in plasma accelerators. Supporting data for our experiment have been provided in the “Supplementary Material”, where the angularly integrated radiation spectra measured with independent diagnostics have been compared with those resulting from the fitting procedure, showing good agreement. In the same file, we have also provided more information on the transmission curves characterizing the absorption mask.

## Results

## Experimental results

**Figure 3:**
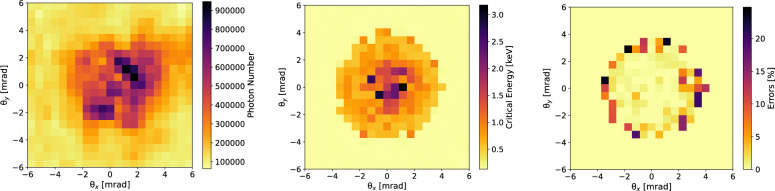
Reconstructed photon number density per bin (left) and measured distribution of the critical energy of the synchrotron-like radiation spectrum (center). Angular distribution of the fit errors on the critical energy (right).

Figure 3 shows, on the left, the measured photon number per bin on the X-ray detector, obtained by summing the pixel values in each of the absorption matrix element, accounting for the different absorptions in the micropattern. At the center of Fig. 3 we report the fitted angular map of critical energies. For a radiation spectrum that is not exactly equal to synchrotron radiation, the critical energy is the one that divides the spectrum into two parts each carrying the same amount of energy. The left plot in Fig. 3 is only needed for a better interpretation of the central plot, to visualize where the most of the photons are. However, only the central plot is needed for the reconstruction of the lateral coherence function, containing the information on how the field amplitude and phase are distributed in the frequency domain, both determining the local spectrum. The method used for the fit is reported and detailed in the “Methods” section. The lower detection threshold of the method was 0.3 keV. Every value below such a threshold was badly retrieved, yielding 0.3 keV. In the peripheral areas of the betatron radiation beam, retrieval of the spectral-angular features is more difficult due to noise and, furthermore, photon spectra are red-shifted towards lower energies. This explains why the central plot in Fig. 3 shows a flattened peripheral region of critical energy: it is here that the reconstruction algorithm fails, but also this is the region where almost no photons are emitted (therefore, the most influenced by the noise). Moreover, the apparently sharp transitions within the map are due to the limited resolution ($\gtrsim 0.1$ keV) of the method in measuring the critical energy.

The errors on the critical energies have been evaluated summing under square root the $\chi ^2$-test error related to the fitting procedure and the error related to the thickness of the filters. However, the most relevant contribution to the error is given by the fitting procedure. The error is quantitatively shown in Fig. 3 (right).

**Figure 4:**
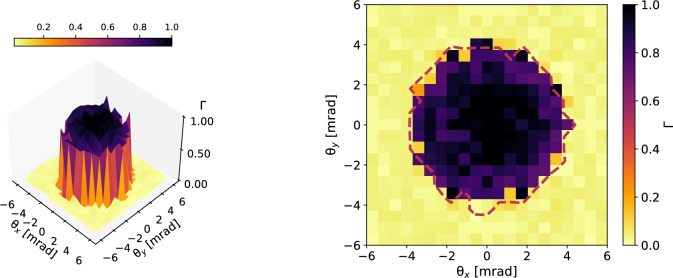
Top: the 3D plot of the measured lateral coherence. Bottom: the corresponding 2D projection with the contour line in red dashed color at $\Gamma =0.5$, determining the coherence area $A_c$.

The angular map of critical energies reveals a nontrivial spectral-angular correlation with hot-spots and asymmetric patterns that are impossible to explain by the on-axis relativistic Doppler effect. A 1D synchrotron-like model for betatron radiation would have predicted an azimuthally symmetric angular map of critical energies^11,21,31^. In fact, the phase of the radiation field in the proximity of a turning point of the oscillating trajectory can be found as^11^:

4 $$\begin{aligned} \vec {H}(\theta ,\phi ,t)\propto e^{i \omega t \left[ \overbrace{1-\frac{1}{c}\frac{dz}{dt} \cos {\theta }}^{On\text {-}Axis\ Doppler\ Effect}+\overbrace{\frac{\omega _\beta }{c}\sin {\theta }(x_\beta \cos {\phi }\sin {\psi _x}+y_\beta \sin {\phi }\sin {\psi _y})-\frac{ \omega ^2_\beta }{4c^2}\sin {\theta }\left( x_\beta ^2\cos {2\psi _x}+y_\beta ^2\cos {2\psi _y}\right) }^{Betatron\ Phase\ Modulation}\right] } \end{aligned}$$

where the radiation is mostly emitted at the turning points of the betatron trajectories^11,31^. The $\psi$-phases are related with the initial transverse momentum^26^ of the electrons at the injection in the plasma accelerating cavity (also called bubble), $x_\beta ,y_\beta$ are the amplitudes of the betatron oscillations in the two transverse planes, $\omega _\beta$ is betatron frequency, and *c* is the speed of light in vacuum. The phase modulation imposed by the betatron motion breaks the azimuthal symmetry of the source. To understand the above statement, is necessary to notice that the phase term in Eq. (4), under the label “On-Axis Doppler Effect”, is only $\theta$-dependent, while the second term depends both upon $\theta$ and $\phi$. The first term can be seen as approximately linear in *t* (due to the fact that $z\simeq v_z t$, where $v_z$ is the velocity of the electron along the acceleration axis): the frequency spectrum of the field achieves an upward shift proportional to $1/( 1-v_z \cos {\theta }/c)$, which goes by the name of relativistic Doppler effect and is azimuthally symmetric. It’s worth specifying that Eq. (4) omits another phase term proportional to the third power of *t*^11^. In the synchrotron-like radiation limit, the cubic term determines the well-known expression for the critical frequency $\omega _c\propto 1/(1+\gamma ^2 \theta ^2)^{3/2}$, where the Lorentz factor is $\gamma \simeq 1/\sqrt{1-v_z^2/c^2}$, which is notoriously azimuthally symmetric^11,21,31^. Therefore, our argument is that if the spectral-angular correlation of a betatron source is not azimuthally symmetric, this must be due to a non-negligible contribution from the betatron phase modulation. Indeed, the second term of phase in Eq. (4) corresponds to a spatial phase modulation, since it deforms the otherwise spherical wavefront of the radiation field. Moreover, it is called “Betatron Phase Modulation” because it strongly depends upon betatron parameters as the oscillation amplitude and phase. This effect is purely three-dimensional and connected with the lateral coherence function introduced as Eq. (3). A numerical demonstration of the above arguments is provided the “Methods” section (“Validation with synthetic data” subsection). The consideration of a betatron phase modulation appears necessary for data interpretation. A 3D model of the electron dynamics is needed to understand the fine structure of the betatron radiation. In the top of Fig. 4, we report the 3D plot of the reconstructed lateral coherence function $\Gamma$, obtained from the fitted fields $\vec {H}(\theta ,\phi ,\omega )$ at each observation angle, combining them via:

5 $$\begin{aligned} \Gamma (\theta ,\phi )=\frac{\int d\omega \vec {H}^{*}(0,0,\omega ) \cdot \vec {H}(\theta ,\phi ,\omega )}{\sqrt{\int d\omega |H (0,0,\omega )|^2}\sqrt{\int d\omega |H (\theta ,\phi ,\omega )|^2}} \end{aligned}$$

where the point $\theta =0,\phi =0$ individuates the barycenter of the X-ray beam. In the bottom of Fig. 4 is the 2D projection of $\Gamma$. A $\phi$-dependent lateral coherence function may correspond to non-symmetric, local deformations of the wavefront and may strongly affect the value of the coherence area $A_c$ of the source, i.e. its brightness *B* since $B\propto A_c$. The contour line $\Gamma =0.5$ delimits the FWHM coherence area. The mean critical energy from Fig. 3 is measured $E_c=(1.6\pm 0.1)$ keV. The FWHM coherence area is determined as $A^{FWHM}_c=(0.05\pm 0.01)$ msr, corresponding to a rms value of $A^{rms}_c=(0.009\pm 0.002)$ msr. The error has been evaluated using the van Cittert–Zernike theorem for which $A_c\propto 1/\sigma _x\sigma _y$, propagating the errors on the source size. Even if the van Cittert–Zernike theorem is normally expressed for monochromatic radiation, the scaling with the source size holds true also for broadband sources. We have observed that applying the reconstruction algorithm without considering the electron acceleration, the measured coherence area would have been underestimated by 4%, for the mean critical energy would have increased and the coherence area would have decreased. Indeed, neglecting acceleration leads to an underestimation of the low-frequency spectrum^31^. Thus, for applications requiring precise analysis and control of lateral coherence, such as imaging relatively large samples, particle acceleration cannot be neglected. Coherence area provides a measure of the area of a sample that can be irradiated and imaged using phase contrast techniques for a fixed source-to-object distance and is related to the most important parameter of a light source: its brightness.

### 2D electron beam emittance

The double information on the spectral and angular distribution of the betatron radiation allows to recover information on the electron beam in both planes of betatron oscillation^28^.

**Figure 5:**
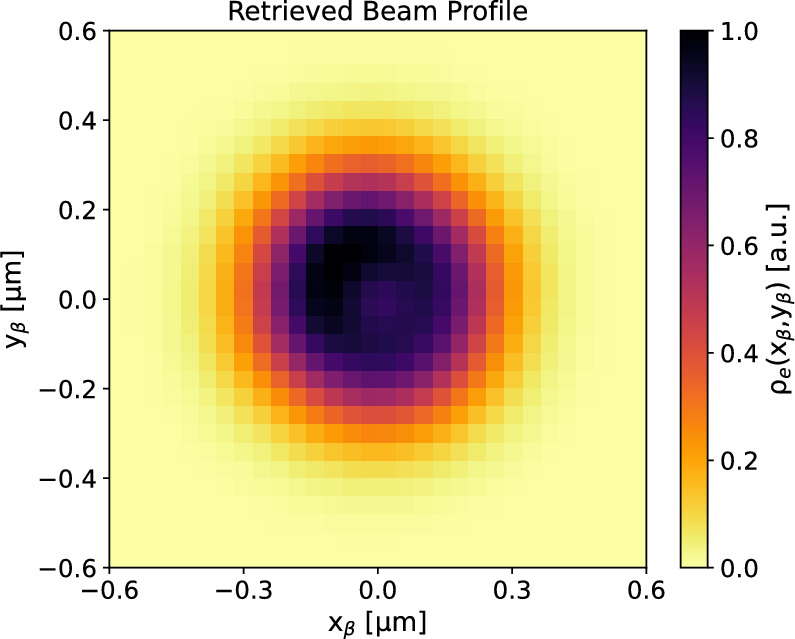
Transverse beam profile of the X-ray source. The particle density $\rho _e(x_\beta ,y_\beta )$ is reported in arbitrary units.

In fact, the spectral-angular distribution of the radiation is linked to the profile of the electron beam in the plasma channel through the distribution of the betatron oscillation amplitudes, i.e. the profile of the X-ray beam on the source plane, which mainly corresponds to the last stage of the acceleration process where the energy of the electrons is maximum (due to the power law scaling between X-ray yield and electron energy^31^). The size and shape of the betatron radiation source is comparable to that of the electron beam within the plasma channel. Figure 5 depicts the transverse profile of the betatron X-ray sources $\rho _e(x_\beta ,y_\beta )$, as retrieved from the data fit. This profile corresponds to the histogram of the betatron oscillation amplitudes $x_\beta ,y_\beta$ used to find the fitting radiation field (see Eq. (4)), integrating over all the observation angles. The FWHM size of the X-ray source at the end of the acceleration channel is $\sim$ 0.4 $\upmu$m in both planes, which, for the scaling^31^ $x_\beta ,y_\beta \propto 1/\gamma ^{1/4}$, corresponds to an estimated beam size at the injection plane of $\gtrsim 1\,\upmu$m. Given a mean electron energy of about 240 MeV, we find that only a sub-micrometric value for the beam size value can explain the observed X-ray spectrum, which extends over a few keV with a critical energy below 2 keV. Sub-micrometric sources have been already demonstrated in literature^30,32–34^. In particular, the rms size of the X-ray source (and thus of the electron beam) is measured as $\sigma _x=(0.17\pm 0.02)\,\upmu$m and $\sigma _y=(0.18\pm 0.02)\,\upmu$m for the horizontal and vertical plane, respectively. Using the formulas for the uncorrelated normalized rms $x-y$ emittance, i.e. $\varepsilon _x={\bar{\gamma }}\sigma _x\theta _x$ and $\varepsilon _y={\bar{\gamma }}\sigma _y\theta _y$, it is possible to obtain an indirect measure of the emittance of the 2D electron beam. For the angular divergences we consider the values $\theta _x=(3.0\pm 0.3)\ mrad,\theta _y=(2.8\pm 0.3)\ mrad$ measured from the betatron radiation beam in Fig. 3 (left plot). In fact, the electron divergence of a self-injected beam is determined by the betatron motion and it corresponds to the betatron beam divergence^5,21,31,34^. In this work $\varepsilon _x=( 0.24\pm 0.04)\,\upmu$m and $\varepsilon _y=(0.24\pm 0.04)\,\upmu$m. Corrections for the relatively large energy spread of the electron beam^35^ would lead to the estimation of larger and more uncertain emittance values as $\varepsilon _x=( 0.29\pm 0.06)\,\upmu$m and $\varepsilon _y=(0.29\pm 0.06)\,\upmu$m. An analysis including phase-space correlation^34^ would yield a negative correction, therefore lower mean values of emittance, but higher uncertainty related to the correlation model. Since most of the radiation is emitted at the turning points of the betatron trajectories, the emittance measured by betatron radiation is predominantly uncorrelated. Furthermore, the information provided by betatron radiation refers mainly to the final stage of the acceleration, where the energy of the electrons is maximum. In fact, the radiation power scales non-linearly with the electron energy^31^. Any of the emittance degradation effects at the plasma’s outlet cannot be measured by betatron radiation, as these occur when the emission process has already ended.

## Discussion

In summary, we observed the effect of a nontrivial spatial phase imposed on the radiation field by the betatron trajectories, determining lateral coherence and brightness of the source. The lateral coherence function was retrieved. To interpret the experimental results, we adopted a time-dependent 3D model of the wiggler-like electron dynamics. Particle acceleration increases the lateral coherence of the X-ray source. With a minimization algorithm, we were able to measure the size of the X-ray source in the two emission planes, paving the way for the single-shot measurement of the 2D normalized rms emittance of the electron beam^28,34^. The time required to reconstruct a beam profile was about 1 h, a time that can be improved by an order of magnitude through parallel computing, making it more feasible as online beam diagnostics in plasma accelerators. The resolution with which the emittance can be determined is not better than that found in previous works^30,34^, but provides, for the first time, the possibility of a bidimensional recovery of the information, i.e. of the complete transverse emittance of the accelerated beams. The provided methodology may be of paramount importance for user applications of novel X-ray sources^25^. The amount of data obtained by means of the proposed methodology allows estimating the peak brightness of the betatron source as $B=N^{0.1\%bw}_{ph}A_c/ 4\pi ^2\theta _x\theta _y \tau \lambda ^2_c\simeq 10^{20}$ ph/s mm$^2$ mrad$^2$ 0.1%bw, where $\tau \simeq 33$ fs, $\lambda _c=0.78$ nm corresponds to the critical energy of 1.6 keV and $N^{0.1\%bw}_{ph}$ is the photon number within a $0.1\%$ relative bandwidth centered at 1.6 keV.

## Methods

### Laser-plasma accelerator

**Figure 6:**
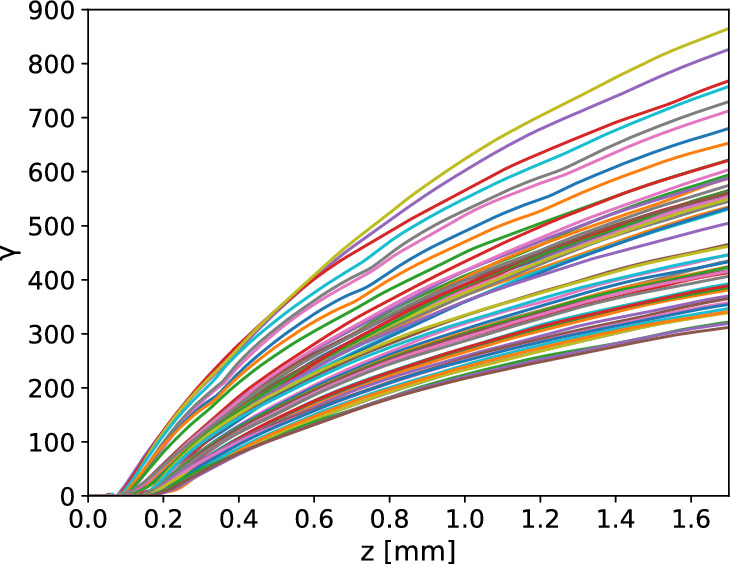
Energy gain of electrons accelerated in the laser-generated plasma bubble, as simulated by FBPIC.

Referring to Fig. 1, the $150\ TW$ Ti:Sa laser, with a $20\,\upmu$m focal diameter Full Width Half Max (FWHM), interacted with a supersonic He gas-jet creating an electron plasma density of $n_e=(3.8\pm 0.4)\times 10^{18}$ cm$^{-3}$ and an acceleration channel $L_{acc}=(1.7\pm 0.1)$ mm long. The electron plasma density was measured by means of a Mach–Zender interferometer, applying the Abel inversion. The acceleration length was indirectly measured by ratio of the measured mean electron energy to the predicted acceleration field^36^ given the measured laser and plasma parameters. PIC simulations confirmed this value, showing that the self-injection of electrons takes place after the vacuum-plasma interface and that the acceleration to the observed energies requires about 1.7 mm (see Fig. 6). The spectrum of the electron beam accelerated via the Laser WakeField Acceleration (LWFA) mechanism, realized in the self-injection regime^37^, was measured by a magnetic spectrometer. The final mean electron energy was 240 MeV (mean Lorentz factor ${\bar{\gamma }}=470$) with a relative rms (root mean square) spread of $20\%$, corresponding to an accelerating field $E_{acc}\simeq 140$ GeV/m. Betatron radiation was produced by the electrons wiggling in the plasma accelerating cavity (see bottom-left inset of Fig. 1, where a quasi-3D FBPIC^38^ simulation is shown).

### Theoretical background

The theoretical description underlying the fitting procedure of this work started from considering electron oscillating trajectories of the following kind:

6 $$\begin{aligned}{} & {} x(t)=x_\beta (t)\cos {\left( \int _{0}^t \omega _\beta (t') dt'+\psi _x\right) }\nonumber \\{} & {} y(t)=y_\beta (t)\cos {\left( \int _{0}^t \omega _\beta (t') dt'+\psi _y\right) } \nonumber \\{} & {} z(t)\simeq \int _0^t\left[ 1-\frac{1}{2\gamma ^2(t')}-\frac{1}{2 c^2}\left( \frac{d x}{dt'}\right) ^2-\frac{1}{2 c^2}\left( \frac{d y}{dt'}\right) ^2\right] dt' \end{aligned}$$

We verified that the electron trajectories calculated by the PIC simulation where well represented and fitted by Eq. (6). We chose a description in terms of an adiabatic motion, i.e. for oscillation amplitudes $x_\beta (t),y_\beta (t)\propto \gamma ^{-1/4}(t)$^31^ and a betatron frequency $\omega _\beta (t)\propto \gamma ^{-1/2}(t)$, slowly varying on the scale of a betatron period. This was already demonstrated to be a valid approximation for the case of relatively long plasma channels^31^.

Equation (6) are solutions for electron dynamics that can be found directly from the wakefield potential of the plasma bubble^31^. The time dependence of the Lorentz factor was chosen to be non-linear, to account for dephasing effects that might affect the acceleration of some electrons. Figure 6 shows the energy gain of electrons accelerated in the plasma bubble, simulated via the quasi-3D FBPIC code. The curvature of the Lorentz factor suggested that a model including acceleration dephasing was suitable for the studied case, even if the radiation is mostly emitted at the final stage of the acceleration process and the curvature of the energy gain turns to be a minor effect. The distribution of the final electron energy at $z=1.7$ mm agreed well with the spectral distribution reported in Fig. 1. A large amount of electrons are accelerated to energies around 250 MeV (corresponding to ${\bar{\gamma }}\sim 500$). Furthermore, a significant fraction of electrons populates the low energy tail of the spectrum, down to $\sim 100$ MeV. Finally, the high-energy tail above 300 MeV is scarcely populated. Every electron emits radiation due to the oscillatory motion described by Eq. (6). The magnetic field of radiation far from the source is found as exact solution of Maxwell’s equations:

7 $$\begin{aligned} \vec {H}(\theta ,\phi ,\omega )=\frac{i\omega }{4 \pi c}\frac{e^{i\frac{ \omega R}{c}}}{R^2}\vec {R}\times \widetilde{\vec {j} }(\vec {k},\omega ) \end{aligned}$$

where $\vec {R}=R(\cos {\phi }\sin {\theta }{\hat{x}}+\sin {\phi }\sin {\theta }{\hat{y}}+\cos {\theta }{\hat{z}})$ is the observation direction, *R* being the source to detector distance, $\omega$ is the photon frequency ($\vec {k}=\omega {\hat{R}}/c$). The Fourier-transform of the current density is^39^:

8 $$\begin{aligned} {\widetilde{j}}_x= & {} \frac{q}{4\pi }\sum \limits _{\alpha ,n,\mu ,\nu }\int _{0}^t x_\beta \omega _\beta \left( J_{\alpha +2\mu }\left( \rho _x^{(1)}\right) \left[ J_{n-\alpha +2\nu +1}\left( \rho _y^{(1)}\right) \right. \right. \nonumber \\{} & {} \left. \left. +J_{n-\alpha +2\nu -1}\left( \rho _y^{(1)}\right) \right] J_{\mu }\left( \rho _x^{(2)}\right) J_{\nu }\left( \rho _y^{(2)}\right) R_n e^{-i \alpha \psi _x-i(n-\alpha ) \psi _y}\right) dt' \nonumber \\ {\widetilde{j}}_y= & {} \frac{q}{4\pi }\sum \limits _{\alpha ,n,\mu ,\nu }\int _{0}^t y_\beta \omega _\beta \left( J_{\alpha +2\mu }\left( \rho _x^{(1)}\right) \left[ J_{n-\alpha +2\nu +1}\left( \rho _y^{(1)}\right) \right. \right. \nonumber \\{} & {} \left. \left. +J_{n-\alpha +2\nu -1}\left( \rho _y^{(1)}\right) \right] J_{\mu }\left( \rho _x^{(2)}\right) J_{\nu }\left( \rho _y^{(2)}\right) R_n e^{-i \alpha \psi _x-i(n-\alpha ) \psi _y}\right) dt'\nonumber \\ {\widetilde{j}}_z= & {} \frac{q c}{2\pi }\sum \limits _{\alpha ,n,\mu ,\nu }\int _{0}^t J_{\alpha +2\mu }\left( \rho _x^{(1)}\right) J_{n-\alpha +2\nu }\left( \rho _y^{(1)}\right) J_{\mu }\left( \rho _x^{(2)}\right) J_{\nu }\left( \rho _y^{(2)}\right) R_n e^{-i \alpha \psi _x-i(n-\alpha ) \psi _y} dt' \end{aligned}$$

with $\rho _x^{(1)}=\omega x_\beta \sin {\theta }\cos {\phi }/c$, $\rho _y^{(1)}=\omega y_\beta \sin {\theta }\sin {\phi }/c$, $\rho _x^{(2)}=\omega \omega _\beta x^2_\beta \cos {\theta }/8 c^2$, $\rho _y^{(2)}=\omega \omega _\beta y^2_\beta \cos {\theta }/8 c^2$. The electron charge is *q*, while the time-dependent resonance function is:

9 $$\begin{aligned} R_n(t)=\frac{i-ie^{i\left[ \left( \left\langle \frac{ 1+\gamma ^2\theta ^2}{2 \gamma ^2}\right\rangle _t + \frac{ K_\beta ^2(t)}{4 \gamma ^2(t)}\right) \omega -n \left\langle \omega _\beta \right\rangle _t \right] t}}{\left( \left\langle \frac{ 1+\gamma ^2\theta ^2}{2 \gamma ^2}\right\rangle _t + \frac{ K_\beta ^2(t)}{4 \gamma ^2(t)}\right) \omega -n \left\langle \omega _\beta \right\rangle _t } \end{aligned}$$

We defined the time-averages as $\langle f \rangle _t=\int _{0}^{t}f(t')dt'/t$. For the experimental conditions explored in this article the so-called strength parameter $K_\beta = \gamma r_{\beta } \omega _\beta /c$ (with $r_\beta ^2=x_\beta ^2+y_\beta ^2$), although it depends on the oscillation amplitudes of the electrons, could not be considered, on average, much greater than one. Due to this reason a wiggler-like emission model, as described by Eq. (8), was selected as the most suitable for matching the experimental observations. In the limit of $K_\beta \gg 1$ the proposed model reduces to a 3D synchrotron-like emission model equivalent to that in Ref.^40^ and for the 1D case it reduces to the undulator model in Ref.^21^. Via Eq. (8) it was possible to calculate the radiated energy *dE* per unit frequency $d\omega$ and solid angle $d\Omega$^21^:

10 $$\begin{aligned} \frac{d^{2} E}{d\omega d \Omega }=\frac{ \omega ^2}{ \pi \varepsilon _0 c^3}\left[ {\widetilde{j}}_x^2+{\widetilde{j}}_y^2+{\widetilde{j}}_z^2 \theta ^2 - 2{\widetilde{j}}_z({\widetilde{j}}_x \cos {\phi }+{\widetilde{j}}_y\sin {\phi })\theta \right] \end{aligned}$$

It should be noted that acceleration and 3D motion were included in the radiation field for the wiggler radiation regime, similarly to what was done in Ref.^40^ for the synchrotron radiation regime. Finally, the total radiation spectrum emitted by a beam of electrons was calculated as an average of Eq. (10) upon the transverse particle distribution $\rho _e(x_\beta ,y_\beta )$.

### Validation with synthetic data

Prior to the experiment we developed a numerical code for the analysis of raw data of the kind shown in Figs. 1 and 2, able to fit the local X-ray absorption pattern across the transmission mask with a suitable radiation field. The radiation field was fitted using Eqs. (6), (7), (8), (9), (10).

For the generation of the synthetic data we considered a bi-gaussian electron beam with final rms size equal to $0.3\, \upmu$m in the horizontal plane and $0.2\, \upmu$m in the vertical plane, with the same energy spectrum as the beam of the experiment. The azimuthal asymmetry was specially chosen to explore the capabilities of the reconstruction algorithm. Once the radiation from the input beam was calculated, the transmission pattern was evaluated directly after, in order to simulate synthetic raw data of the type in Fig. 1. Such transmission pattern was obtained accounting for the transmission function of the detection system (X-ray filters, detector), introduced in Fig. S2 of Supplementary Material.

**Figure 7:**
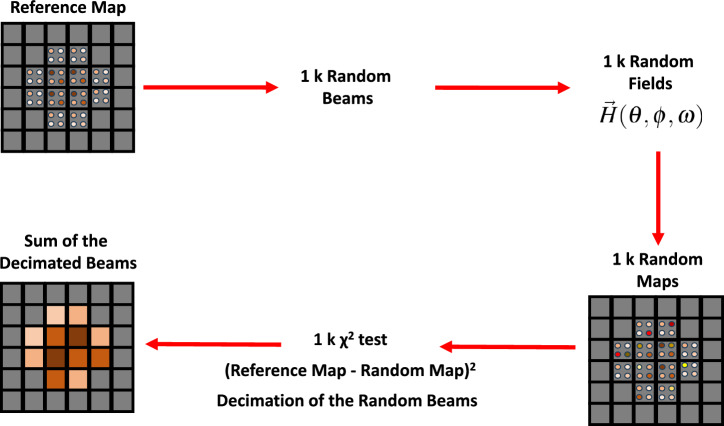
Algorithm for information retrieval.

The validation continued with an information retrieval, based on the algorithm schematized in Fig. 7. In particular, we targeted the retrieval of the spectral-angular distribution of the radiation emitted by the input beam and of its profile, starting from the synthetic transmission pattern, used as a reference map. For the retrieval, for each observation angle, the spectrum emitted by 1*k* random ensembles of electrons was calculated. The resolution at which the solid-angle was sampled was chosen to be the same as the one used in the experiment, namely $1\,\upmu$sr. For the generation of the 1*k* beams, we started creating five random variables $x_\beta (0),y_\beta (0), \gamma (0), \psi _x,\psi _y$ for each electron in an ensemble of 100*k* macroparticles (we verified that increasing the number of macroparticles did not change significantly the results). The betatron oscillation amplitudes $x_\beta$ and $y_\beta$ followed a uniform random distribution centred in zero, the width of which was randomly different for each of the test beams in a range from 0 to 1 $\upmu$m, as expected from PIC simulations. The widths of the betatron oscillation amplitude distributions were set equal to three times $c\theta _e/\omega _\beta (z=L_{acc})$^34^, where $\theta _e$ is the measured rms electron vertical divergence, i.e. $\theta _e=(3.3\pm 0.4)\ mrad$ (see Fig. 1).

**Figure 8:**
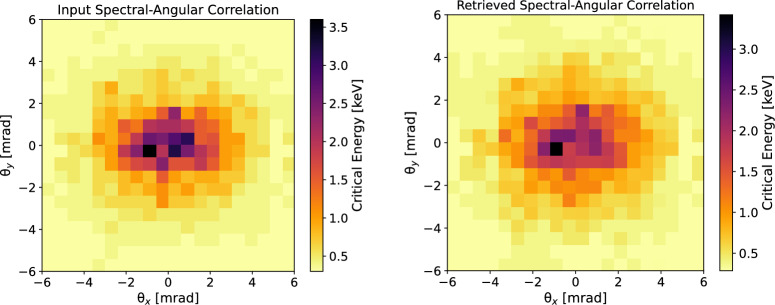
Top: spectral-angular correlation of betatron radiation emitted by the input electron beam used for generating synthetic data. Bottom: retrieved spectral-angular correlation of betatron radiation. The retrieval is achieved by fitting the synthetic data at each observation angle with a radiation field calculated via Eqs. (6), (7), (8), (9).

**Figure 9:**
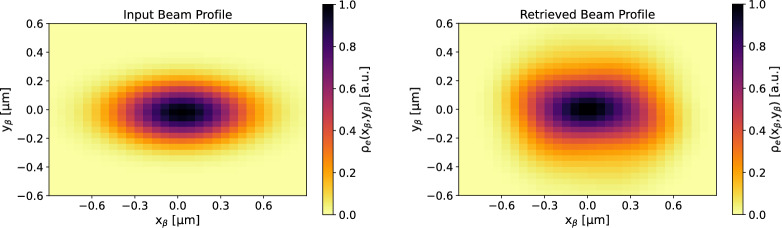
Top: input electron beam profile used for generating synthetic betatron radiation data. The profile is evaluated at the end of the radiation process. Bottom: retrieved electron beam profile at the end of the radiation channel. The retrieval is achieved via the histogram of the betatron radiation amplitudes that best fit the synthetic data at each observation angle.

The initial oscillation phases $\psi _{x,y}$, related to the initial transverse momenta of the particles^26^, were extracted randomly in $[0,2\pi ]$. The initial energies $\gamma (0)$ were distributed around the mean value $\omega _0/\omega _p$^41^ for simulating the random process of self-injection, where $\omega _0$ is the laser frequency and $\omega _p$ the plasma frequency. The initial electron energy distribution was chosen as the measured one, shown in Fig. 1, with the only difference that at $z=0$ the mean value of $\gamma (0)$ was set to $\omega _0/\omega _p$ and the spread to $20\%$ rms, to match the final value of spread at the end of the acceleration process. With this choice, the final spectral shape of the electron energy distribution was preserved to be the same as the measured one. Furthermore, in this way, the path length of each particle was different, since $L\simeq v_z t$ and $v_z \simeq c(1-1/2\gamma ^2)$: the choice to preserve the shape of the measured spectrum for the simulation naturally led to taking into account the different injection times and therefore for a finite bunch length (in this work estimated as $\sim 10\, \upmu$m, corresponding to $\tau =33\ fs$). Therefore, 1*k* test transmission patterns were created for each of the 1*k* random ensembles and each observation angle. The relative difference between input (reference) and test patterns was estimated as the point-by-point root mean square of the differences between the angular transmission maps (called $\chi ^2$ test). Before the $\chi ^2$ test, the reference and test maps were normalized to 1. Only the patterns that differed less than $25\%$ from the input synthetic raw data were stored, discarding the others. This procedure resulted in a decimation of the initial set of 1*k* electron beams. The histogram of the betatron oscillation amplitudes relative to the stored beams, at the end of the procedure, yielded the best reconstruction of the electron beam profile. Furthermore, the sum of the the radiation fields associated with the stored electron beams was used to calculate the angular distribution of the critical energy of the radiation spectrum. Figure 8 shows the spectral-angular distribution of betatron radiation emitted by the input beam chosen to generate synthetic raw data (top) and the retrieval of the same map (bottom). The retrieval was obtained by calculating the critical energy from the best-fitting radiation field at each angle of observation. The correspondence between input and retrieved synthetic data can be considered good. Indeed: the azimuthal asymmetry was well-reproduced and the scale of critical energies is the same. Moreover, integrating over all the angles, the mean critical energy of the radiation emitted by the input beam was 1.7 keV, while the one from the retrieved beam corresponded to 1.8 keV, with a $8\%$ relative error. Figure 9 shows the input beam profile versus the retrieved beam profile. The agreement is rather good, with a $10\%$ relative error in terms of the rms beam sizes. For sake of completeness, in order to fully characterize the precision of our method, we also tested the robustness of the algorithm considering only two different absorption channels in the micro-pattern instead of three. We considered the absorption in the $20\, \upmu$m and $40\,\upmu$m channels, discarding the absorption in the $60\,\upmu$m Mylar layers. We didn’t notice a significant difference. In fact, the beam symmetry and sizes were reconstructed with a precision that was worse by less than $2\%$ compared to the case with three absorption channels. This evidence allowed us to eliminate any doubt that the micro-pattern on the transmission mask was not sufficient for the reconstruction of the spectral-angular characteristics of the betatron radiation source during the experiment. In principle, it may seem that the accuracy of the reconstruction algorithm can be increased by setting tighter limits to the $\chi ^2$ test. In practice, this does not happen for two reasons: setting stricter limits would mean storing fewer electron beams, but this could lead to the undesirable result of an even worse retrieval. To avoid the latter case, one should increase the number of test electron ensembles, but this could significantly slow down the procedure, so it is not practically feasible.

**Table 1:** Rms beam sizes evaluated on the retrieved beam profiles obtained after different runs of the reconstruction algorithm.

Simulation N$^{o}$	Retrieved $\sigma _x$ ($\upmu$m)	Retrieved $\sigma _y$ ($\upmu$m)
1	0.31	0.21
2	0.30	0.21
3	0.30	0.19

**Figure 10:**
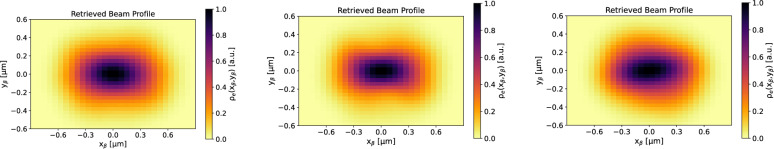
Reconstructed beam profiles for different runs of the reconstruction algorithm.

The resolution of the method was determined by a study of the stability of the reconstruction algorithm on different implementations for the same case study of Fig. 9. Indeed, we conducted several campaigns of beam profile reconstruction (three of them reported in Fig. 10). The solution for the beam profile provided by the reconstruction algorithm showed to be quite stable. Indeed, the error of the method was $1\,\upmu$m around $20\,\upmu$m and only differences of $2\,\upmu$m could be resolved by the proposed algorithm (see Tab. 1, where the values of beam size obtained from the reconstructed profiles of Fig. 10 are reported).

**Figure 11:**
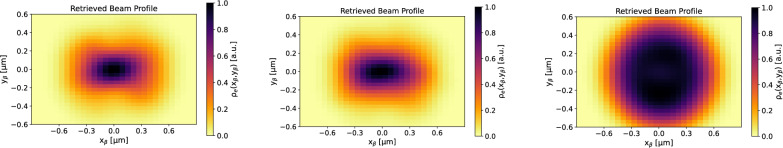
Reconstructed beam profiles for different runs of the reconstruction algorithm at different threshold for the $\chi ^{2}$-test between the input transmission pattern and the test patterns (from left to right: $\chi ^{2}<20\%$, $\chi ^{2}<25\%$, $\chi ^{2}<30\%$).

Such a resolution was considered acceptable for the experiment. Thus, the algorithm of retrieval presented in this section was adopted to analyze the experimental data reported in the “Results” section. Figure 10 shows that there is no unique solution for the beam reconstruction (and analogously for the lateral coherence function), but a set of solutions with similar statistical properties. In general, the $\chi ^2$ minimization is not a sufficient condition to conclude that the free parameters used for minimization are the best parameters. However, the accuracy on the reconstructed beam size reached during the validation phase was assumed to be the same during the experiment. This could be motivated by the fact that the order of magnitude of the key parameters (critical energy, beam size and energy spectrum) was the same in the two cases, so there was no reason to believe that the method could behave in a radical different way for the two cases. Indeed, the synthetic data were generated starting from an input beam with the same energy spectrum as the beam of the experiment and with a beam size (around $0.2\,\upmu$m rms) that could justify the measured critical energy. We achieved, on synthetic data, a reconstruction of the lateral coherence function and of the beam profile with an uncertainty around $10\%$. This brought us to consider our retrieval strategy robust A further confirmation that the threshold for the $\chi ^{2}$ used for the reconstruction was adequate, was given by a study of the impact of it on the beam profile reconstruction of synthetic data. Indeed, Fig. 11 shows that the threshold value $25\%$, of difference between input and test absorption pattern, was adequate for the purpose of beam reconstruction. A lower threshold (20%) could also be adequate but required more test beams (for the left of Fig. 11 we considered 2*k* test beams at each iteration). A higher threshold (30%) would have been such to prevent the convergence of the method to an acceptable result.

**Figure 12:**
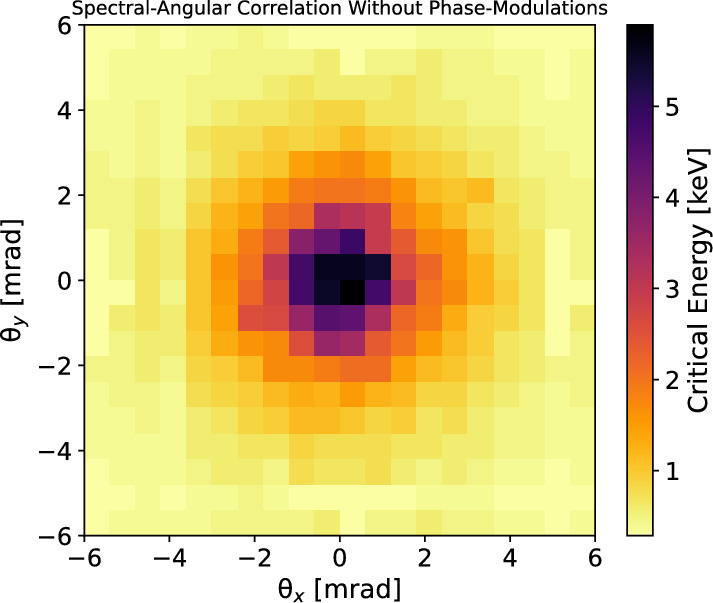
Spectral-angular correlation of betatron radiation calculated neglecting betatron phase modulations.

Before concluding, we would like to show the reconstruction of the spectral-angular correlation of the radiation emitted by the input beam if neglecting spatial phase modulations, i.e. $\rho _x^{(1)},\rho _y^{(1)}\sim 0$ in Eq. (8). From Fig. 12 is possible to see that when neglecting the betatron phase contribution in the radiation field, two fundamental features of the source are not well-reproduced: (1) the azimuthal asymmetry of the spectral-angular correlation, (2) the mean value of the critical energy. The result is significantly different from Fig. 8, and wrong.

Thus, we observe that betatron phase modulations are responsible for the nontrivial spectral-angular distribution of a betatron source. Neglecting the local red-shifts imposed by the violent betatron oscillations, leads to a significant overestimation of the average critical frequency expected from the source and to an improper simplification of the spectral-angular correlations of the photon beam. The latter are important for the design of betatron X-ray sources, as well as for extrapolating information from spectral data.

### Supplementary Information

Supplementary Information.

## Data Availability

The datasets used and/or analysed during the current study available from the corresponding author on reasonable request.
