# Supplementary material for: Reconstruction of lateral coherence and 2D emittance in plasma betatron X-ray sources
Source: Sci Rep. 2024 Jan 19;14:1719. doi: 10.1038/s41598-024-52231-z (PMC10799011; doi:10.1038/s41598-024-52231-z)
Supplement: Supplementary file 1 — Supplementary Information. [file 41598_2024_52231_MOESM1_ESM.pdf]

# Lateral coherence and phase modulations of plasma betatron X-ray sources: Supplementary Material

Alessandro Curcio

October 4, 2023

## 1 Supporting Data

The angularly-integrated betatron radiation spectrum is shown in Fig. S1 (dashed colored lines). In particular, five different measurements of betatron radiation spectra are reported (thin colored dashed lines) which have been detected during the experiment by means of a X-ray CCD camera. The camera used was a Great Eyes 1024 256, with an image area of 26.6 $mm \times 6.7$ $mm$, a pixel size of 26 $\mu m \times 26$ $\mu m$, operated at a pixel readout frequency of 500 $kHz$ and a sensor temperature of $-20°$. The betatron radiation spectrum obtained

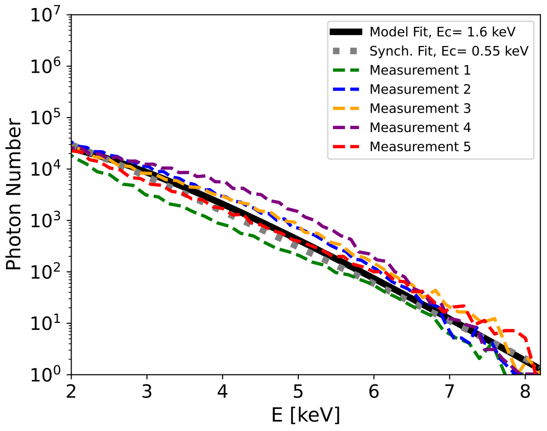


Figure S1: Comparison of angularly-integrated measured spectra with synchrotron-like spectral model ($\propto E^2 K^2_{2/3}(E/2E_c)$) at critical energy $E_c = 0.55$ $keV$ and with the fitting model of this paper (Eq. 4 in the Main Manuscript), corresponding to a critical energy of 1.6 $keV$.

via an angular integration of the fitted radiation field (continuous black line), corresponding to Fig. 2 in the Main Manuscript, is in good agreement with the independent measurements obtained with the CCD camera. Conversely, model-

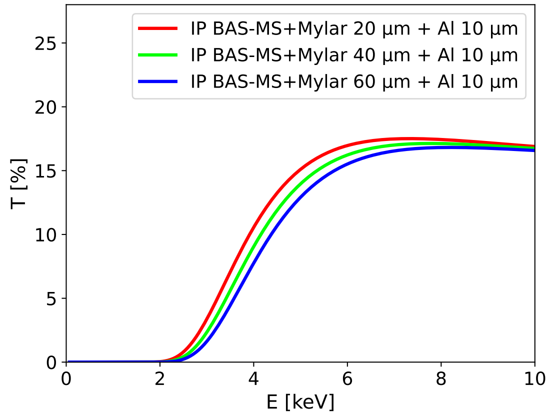


Figure S2: Transmission curves of the diagnostic apparatus. The betatron radiation spectrum emitted by the electrons accelerated in the plasma bubble is attenuated by the 10 $um$ Al foil wrapping the IP detector, and by the Mylar transmission channels of the absorption mask. The attenuation is strong below 2 $keV$. The transmitted radiation is detected and analyzed with the undulator model described by Eq. 4 in the Main Manuscript.

ing the system by means of a 1D synchrotron-like model [1,2,3] would have given a critical energy below 1 $keV$ (thick gray The maximum strength parameter for the experiment in this work is found as $K_\beta = \gamma r_\beta \omega_\beta / c \lesssim 3$. This parameter characterizes the regime of emission and only values much larger than one would allow the use of a synchrotron-like description of the betatron radiation [1]. In fact, it is known that adopting the synchrotron-like approximation for the case of $K_\beta \gtrsim 1$, while it should be used for $K_\beta >> 1$, can lead to a serious underestimation of the critical energy [4]. For $K_\beta \gtrsim 1$, the regime of emission is wiggler-like, described by Eq. 4 in the Main Manuscript. The largest part of the radiation intensity falls below 2 $keV$, yielding a total estimated photon number per shot around $N_{ph} \sim 10^8$. However, radiation below 2 $keV$ is not directly detected because it is strongly attenuated by the filtering system of the diagnostic apparatus, made up of the Al foil that wraps the IP detector and the Mylar thicknesses of the absorption mask. The fitting procedure involves only the high-frequency spectrum, exactly as in other X-ray diagnostic techniques that make use of absorption foils acting as high-pass filters. According to Fig. S2 , the maximum sensitivity of the measurement is obtained around $4-5$ $keV$ where the thickest absorption channel absorbs about twice as much as the thinnest layer. The angular resolution may be increased by two orders of

magnitude if a filter with a smaller pattern is used (in this work each individual absorption channel in the filter had a diameter of 500 $\mu m$).

## 2 Bibliography

1. Esarey, E., Shadwick, B., Catravas, P. & Leemans, W. Synchrotron radiation from electron beams in plasma-focusing channels. Phys. Rev. E 65, 056505 (2002).

2. Corde, S. et al. Femtosecond x rays from laser-plasma accelerators. Rev. Mod. Phys. 85, 1 (2013).

3. Thomas, A. G. R. Scalings for radiation from plasma bubbles. Phys. Plasmas 17 (2010).

4. Lau, Y., He, F., Umstadter, D. P. & Kowalczyk, R. Nonlinear thomson scattering: A tutorial. Phys. Plasmas 10, 2155–2162 (2003)
